# Quantitative proteomic analysis in HCV-induced HCC reveals sets of proteins with potential significance for racial disparity

**DOI:** 10.1186/1479-5876-11-239

**Published:** 2013-10-01

**Authors:** Simon T Dillon, Manoj K Bhasin, Xiaoxing Feng, David W Koh, Sayed S Daoud

**Affiliations:** 1 Beth Israel Deaconess Medical Center (BIDMC), Harvard Medical School, Boston, MA USA; 2 Department of Pharmaceutical Sciences, Washington State University, Pullman, WA USA

**Keywords:** Hepatocellular carcinoma, Hepatitis C, Tissue proteomics, Isobaric tags for relative and absolute quantification (iTRAQ), Cancer racial disparity

## Abstract

**Background:**

The incidence and mortality of hepatitis C virus (HCV)-induced hepatocellular carcinoma (HCC) is higher in African Americans (AA) than other racial/ethnic groups in the U.S., but the reasons for this disparity are unknown. There is an urgent need for the discovery of novel molecular signatures for HCV disease progression to understand the underlying biological basis for this cancer rate disparity to improve the clinical outcome.

**Methods:**

We performed differential proteomics with isobaric labeling tags for relative and absolute quantitation (iTRAQ) and MS/MS analysis to identify proteins differentially expressed in cirrhotic (CIR) and HCC as compared to normal tissues of Caucasian American (CA) patients. The raw data were analyzed using the ProteinPilot v3.0. Searches were performed against all known sequences populating the Swiss-Prot, Refseq, and TrEMBL databases. Quality control analyses were accomplished using pairwise correlation plots, boxplots, principal component analysis, and unsupervised hierarchical clustering. Supervised analysis was carried out to identify differentially expressed proteins. Candidates were validated in independent cohorts of CA and AA tissues by qRT-PCR or Western blotting.

**Results:**

A total of 238 unique proteins were identified. Of those, around 15% were differentially expressed between normal, CIR & HCC groups. Target validation demonstrates racially distinct alteration in the expression of certain proteins. For example, the mRNA expression levels of transferrin (TF) were 2 and18-fold higher in CIR and HCC in AA as compared to CA. Similarly; the expression of Apolipoprotein A1 (APOA1) was 7-fold higher in HCC of AA. This increase was mirrored in the protein expression levels. Interestingly, the level of hepatocyte nuclear factor4α (HNF4α) protein was down regulated in AA, whereas repression of transcription is seen more in CA compared to AA. These data suggest that racial disparities in HCC could be a consequence of differential dysregulation of HNF4α transcriptional activity.

**Conclusion:**

This study identifies novel molecular signatures in HCV-induced HCC using iTRAQ-based tissue proteomics. The proteins identified will further enhance a molecular explanation to the biochemical mechanism(s) that may play a role in HCC racial disparities.

## Background

Hepatocellular Carcinoma (HCC) is one of the few tumors in which the incidence is on the rise worldwide, especially in the United States (US) [1]. The increasing incidence in the US is associated with the rise in hepatitis C virus (HCV) infection [2]. It is estimated that 3.2 million people in this country are infected with HCV; a blood-borne disease linked to 12,000 US deaths a year [3]. More concerning are projections that this rate will quadruple in the next ten years, to over 40,000 cases per year. Although a reliable value for HCV-positive individuals is difficult to determine, the World Health Organization (WHO) estimates that 3% of the world’s population – more than 170 million people – are chronically infected, and that 300 million people are HCV carriers [4]. Following an acute infection with HCV, the disease becomes chronic in about 80% of cases. After 20–30 years of chronic infection, 20-30% of patients develop liver cirrhosis, which leads to HCC in 80-90% of all cirrhotic livers [5].

Inequalities in disease prevalence, treatment, and outcome make HCC an important minority health problem. First, there are disparities in the prevalence of HCV infection with African Americans (AAs) being twice as likely to have been infected compared with Caucasian Americans (CAs) [6]. Additionally, there are significant disparities in access to HCV care for racial/ethnic minorities [7]. Finally, AAs are less likely to respond to anti-HCV therapy than CAs [8], and have a considerably lower likelihood of receiving liver transplantation [9]. Therefore, there is a need for new prognostic markers to understand the molecular mechanisms of HCC disease progression, especially in the presence of cirrhosis, and to establish the precise biological underpinnings of HCC racial disparities. Currently, the most widely used surveillance/diagnostic tests for HCC are ultrasound and serum α-fetoprotein (AFP). The use of ultrasound is particularly subject to low sensitivity and specificity when applied to cirrhotic patients [10]. In addition, serum AFP levels may be normal in up to 40% of patients with HCC, particularly during early stages [11], and elevated AFP have been reported in patients with cirrhosis or exacerbations of chronic hepatitis infection [12,13]. Thus, the use of serum AFP as a diagnostic maker for HCC has multiple limitations when applied to patients with HCV. As a direct consequence of the limitations of these two methods for assessing HCC there exists an urgent need to identify additional biomarkers for prognosis and detection of HCV induced HCC. High-throughput omics technologies have been widely applied, aiming at the discovery of candidate biomarkers for HCC staging, prediction of recurrence and prognosis, and treatment selection. For example, microarray analyses were used to identify genes that are uniquely up- or down-regulated in HCC tissue samples [14-16]. Although these studies have provided important information for elucidating biomarkers for HCC disease progression, they also provided limited diagnostic/prognostic values. In addition, gene expression profiles of most of the discovered genes did not correlate well with changes in protein levels [17]. However, recent advancements in quantitative and large-scale proteomic methods have been used not only for discovery of clinically useful biomarkers for HCC [18], but also in clarifying the molecular mechanisms of disease pathogenesis by using body fluids, such as serum [19,20], and tissue samples [21,22] and cultured cells [23,24].

Since 80% of HCC patients in the US have cirrhosis due to HCV infection, we aimed in the current study to identify tissue protein patterns and differentially expressed protein markers in patients with HCV cirrhosis (HCV+/HCC-) and HCV-associated HCC (HCV+/HCC+) using iTRAQ (Isobaric Tags for Relative and Absolute Quantitation)-based comparative proteomic analyses to assess possible roles of identified proteins in HCC racial disparities. Differential expression of selected, biologically interesting proteins were then validated on two independent sets of liver and tumor tissue samples from AA and CA patients by immunoblot blot (WB) analysis and real-time PCR (qRT-PCR).

## Material and methods

### Ethics statement

The Institutional Review Board at Washington State University (WSU) approved the protocol of the current study. Twenty-six snapped frozen tissue samples (8 included in original analysis and 18 for target validation study) were obtained from the Institutional Research Board (IRB) approved University of Kansas Medical Center Liver Center Tissue Bank. All specimens with anonymized identifiers were histopathologically confirmed by a pathologist.

### Tissue preparation and protein extraction

We studied 9 liver tissue samples from HCV+/HCC + patients, 9 samples from HCV+/HCC- patients, and 8 normal liver samples HCV- de-identified. Relevant clinical information on the patients is shown in Supplemental Table 1. Tissues were prepared as described previously [25]. All tissues were frozen at −80°C until use. Each tissue sample was first frozen in liquid nitrogen and a tissue powder was then generated. The tissue powder was carefully collected and resuspended in RIPA lysis buffer (Boston Bioproducts, Inc. Ashland, MA) supplemented with one tablet of complete protease inhibitor cocktail (Roche) and 1 mM DTT. About 500 ul of lysis buffer was added to each tissue powder and then mixed by vortexing. After centrifugation at 10,000xg for 10 minutes at room temperature, the supernatant was retained as the solubilized whole cell lysate. Retained lysates were acetone precipitated. To 250 ul of whole cell lysate a 6× volume (1.5 ml) of ice-cold 100% acetone was added. Precipitated proteins were then collected by centrifugation at 6,000xg for 10 minutes and pellets were briefly air-dried (1–2 minutes), and then re-suspended in 10 mM TEAB (pH 8.5). After mixing for 1–2 hours at room temperature the re-solubilized proteins were centrifuged at 10,000xg for 10 minutes. The supernatant was retained as the solubilized whole cell lysate used in the iTRAQ assay. Protein concentration of each sample was determined using the Pierce BCA Protein Assay Kit (Thermo Scientific, Rockford, IL). and samples stored in aliquots at −80°C until use.

**Table 1:** Real-time PCR primers

**Gene**	**Primer**	**Sequence**
TF	Forward Primer	5'-ATGAACCAGCTTCGAGGCAA-3'
	Reverse Primer	5'-AGAGGTTTACGTGGCTCAGG-3'
FLNA	Forward Primer	5'-TGTCACAGGTGCTGGCATCG-3'
	Reverse Primer	5-CGTCACTTTGCCTTTGCCTG-3'
APOA1	Forward Primer	5'-CAAGGTCAGCTTCCTGAGCG-3'
	Reverse Primer	5'-CGTTTATTCTGAGCACCGGGAA-3'
18S rRNA	Forward Primer	5'-GTAACCCGTTGAACCCCATT-3'
	Reverse Primer	5'-CCATCCAATCGGTAGTAGCG-3'

### iTRAQ sample labeling

The 8-plex iTRAQ sample protocol (Applied Biosystems, Foster City, CA) was utilized [26]. Briefly, 100 ug of protein from each sample was reduced, alkylated then digested with trypsin, prior to labeling with one of the individual 8-plex-iTRAQ tags (Applied Biosystems, Framingham, MA). The labeled samples were combined, vacuum-evaporated, and stored at −20°C prior to fractionation by strong-cation exchange (SCX) liquid chromatography.

### First dimension separation: strong cation exchange (SCX) chromatography

Half of each pooled iTRAQ labeled sample (400 ug) was added to 1.0 ml of SCX buffer A. After mixing well this was centrifuged at 16,000xg for 5 minutes. The entire sample was loaded with care taken to avoid any pellet at the bottom of the tube. The peptides in the sample were separated using an Agilent 1100/1200 HPLC with a POROS HS/20 column (4.6 mm × 100 mm). Buffer A was 10 mM KH2PO4, 25% acetonitrile (v/v), pH 2.78. Buffer B is Buffer A containing 1 M KCl as described previously [25]. The entire unbound and bound gradient was collected across 96 fractions. Based on the chromatogram, 40 fractions covering all bound and eluted molecules were then run for second dimension separation by LC MALDI-TOF/TOF. Fractions were dried using a vacuum centrifuge as before and each was re-suspended in reverse phase buffer A.

### Second dimension separation: reverse-phase LC-MALDI-TOF/TOF

The 40 SCX fractions were each analyzed by reverse-phase (RP) nanoLC-MALDI-TOF/TOF. Peptides were captured by microflow on an Acclaim PepMap100 C18 cartridge column (500 um i.d. × 5 mm, 5 um, 100A). Peptides were then separated by nanoflow (300 nl/min) over a 15.0 cm long Acclaim PepMap100 C18 column (75 um i.d. × 15 cm, 3 um). Each SCX fraction was printed to ~500 spots per plate with five SCX fractions per plate and CHCA MALDI matrix (5 mg/ml stock solution) mixed in by the Probot just prior to printing. Mass spectrometry was performed on the separated and printed peptides in the 4800Plus MALDI-TOF/TOF Analyzer.

### Mass spectrometry data collection parameters

Peptide MS spectra were captured using the reflector positive mode. The mass window was between 800–4000 Da with the focus mass at 2100. Approximately, 50 laser shots on 20 random spots per printed MALDI spot were collected for a total of 1000 laser shots per spot. The Interpretation Method used for MS/MS peak selection included the top 15 peptides across a three consecutive spot window above a S/N = 70. MS/MS spectra were generated with the CID on (medium pressure) and 50 laser shots on 20 random spots (1000 shots in total) were averaged per fragmented peptide.

The mass spectral data generated were then exported to .txt files. The Peaks to Mascot function in the 4000 Series Explorer™ (AB SCIEX) software was used with the following settings to generate the data files (MS/MS peak filter mass range = 60; precursor = −20; peak density = 5 peaks per 200 Da; Min S/N = 5; Min Area = 50; max peaks/precursor = 40). There were 40 SCX fractions run; therefore, 40 .txt files with the MS and MS/MS peak list data are included in the Exported data.rar (see Additional file 1).

### Bioinformatics analysis

#### Protein identification and relative quantitation

The raw data were analyzed by the ProteinPilot v3.0 software (AB SCIEX) using the Paragon algorithm [27]. Searches were performed against a comprehensive database generated from SwissProt, Refseq and TrEMBL protein sequences. We generated a combined, redundant database of all known human proteins in three publicly available databases. Human proteins from UniProtKB/SwissProt and TrEMBL (http://www.uniprot.org/uniprot/?query=organism:9606+keyword:1185) and the NCBI RefSeq human proteins (http://www.ncbi.nlm.nih.gov/protein) were used and combined into one file containing 121,237 protein sequences. A total of 60,250 MS/MS spectra were generated that were combined and searched against human database file using Protein Pilot 3.0. The sample type was set to iTRAQ 8plex (peptide labeled). Cys alkylation: MMTS; Digestion: trypsin; and the ID focus: Biological modifications and amino acid substitutions. Using these criteria 20,791 spectra (34.3%) were identified with 95% confidence.

The data were normalized for loading error by bias correction and background correction using ProteinPilot 3.0. The confidence value for each peptide was calculated based on agreement between the experimental and theoretical fragmentation patterns. Each protein was provided with a confidence score (0% to 100%) based on confidence scores of its constituent peptides with unique spectral patterns. The proteins with confidence score greater than 90% and with at least 1 peptide of 95% identification confidence were used for further quality control and differential expression analysis. Each protein also achieved quantitative scores for each of the eight-iTRAQ tags to calculate the relative expression levels, as shown in Table 2. In this experiment, the relative expression for proteins in different samples was calculated using a normal sample as the reference sample.

**Table 2:** Differentially expressed proteins (DEP) between cirrhotic HCV + and HCV+/HCC + compared to normal liver tissue samples of Caucasian Americans

**Protein ID**	**Protein name**	**Peptides**	**% Coverage**	**Mean iTRAQ ratios**		
				**Normal**	**HCV+/HCC-**	**HCV+/HCC+**
P00738	Haptoglobin	15	27	1.05	0.19	0.18
P11021	78 kDa glucose –regulated protein	12	23	1.15	0.45	0.18
P01834	Ig kappa chain C region	17	85	1.17	10.65	8.47
P67936	Tropomyosin alpha-4 chain	8	24	0.68	3.40	2.47
Q13011	Delta(3,5)-Delta(2,4)-dienoyl-CoA isomerase	9	26	0.84	0.45	0.47
P62328	Thymosin beta-4	8	77	1.04	4.30	5.68
P05091	Aldehyde dehydrogenase	10	17	1.20	0.37	0.42
Q06278	Aldehyde oxidase 1	5	4	1.06	0.24	0.34
P26038	Moesin	7	15	0.83	2.20	2.02
P54868	Hydroxymethylglutaryl-CoA synthase	7	11	0.87	0.23	0.36
Q09666	Neuroblast differentiation-associated protein	6	5	0.93	3.03	2.78
P23284	Peptidyl-prolyl cis-trans isomerase B	3	13	0.90	0.48	0.37
P34896	Serine hydroxymethyltransferase	4	9	0.89	0.30	0.41
P0CG05	Ig lambda-2 chain C regions	3	35	1.07	20.96	12.90
Q05682	Caldesmon	3	6	0.76	11.04	7.29
*P02647*	*Apolipoprotein A-I*	3	10	1.35	2.79	3.51
*P02787*	*Serotransferrin*	2	3	1.24	6.71	6.30
P00505	Aspartate aminotransferase	2	4	0.86	0.37	0.44
P02741	C-reactive protein	2	3	0.80	0.15	0.13
*P21333*	*Filamin A, alpha*	1	1	0.86	4.15	4.38
P24752	Acetyl-CoA acetyltransferase	7	17	1.05	0.33	0.53
B9A064	Immunoglobulin lambda-like polypeptide 5	3	7	1.47	7.80	6.09
P12532	Creatine kinase B-type	1	6	1.25	8.21	5.36
P63313	Thymosin beta-10	3	64	1.01	2.86	2.46
P02753	Plasma retinol-binding protein	1	5	1.00	3.66	2.82
P01742	Ig heavy chain V-I region	2	10	0.91	5.77	7.12
341914926	PREDICTED: Ig heavy chain V-III region	1	16	1.18	2.89	2.40
Q16555	dihydropyrimidinase-related protein 2 isoform 1	2	3	1.50	3.91	3.02
P13716-2	Isoform 2 of Delta-aminolevulinic acid dehydratase	1	3	0.94	0.49	0.50
Q01995	Transgelin	1	5	1.04	5.20	4.34
P01701	Ig lambda chain V-I region	1	15	0.51	2.31	2.56
P18136	Ig kappa chain V-III region	1	14	1.08	2.22	3.51

Note: Proteins shown in italics were validated using qRT-PCR or WB.

#### Quality control and unsupervised analysis

The quality control analysis was performed on the basis of relative expression values of different proteins to identify any outliers. The quality control analysis was performed using pair-wise correlation plots, boxplots, principal component analysis (PCA) and unsupervised hierarchical clustering. PCA projects multivariate data objects onto a lower dimensional space while retaining as much of the original variance as possible. This is necessary because in analyzing proteomic data, due to a dimensional problem, the number of proteins most often exceeds the number of samples by a considerable amount.

#### Supervised analysis

To identify the differentially expressed proteins (DEP), the relative protein expression values were compared between groups (Normal vs. Cirrhosis (CIR), Normal vs. HCC, Cirrhosis vs. HCC). Proteins were considered overexpressed in HCC compared to normal if the iTRAQ ratio of HCC compared to Normal was greater than 2.0, and if the corresponding maximum normal to normal ratio was less than the HCC to normal ratio. Similarly, proteins were considered under-expressed in HCC relative to normal if the iTRAQ ratio of HCC to normal was less than 0.5 and if the corresponding minimum normal ratio was higher than the HCC to normal ratio. Using the same method, DEP were identified for CIR vs. normal, and HCC vs. CIR comparisons.

In order to identify proteins patterns that are specifically differentially expressed in HCC or HCC and CIR as compared to normal and functionally related, we performed self-organizing map (SOM) analysis on the differentially expressed proteins identified as described in the previous section. We carried out SOM clustering on relative protein expression values using Pearson correlation coefficient based distance metrics and a target of 9 groups. SOM allow the grouping of protein expression patterns into an imposed structure in which adjacent clusters are related, thereby identifying sets of proteins that follow certain expression patterns across different conditions.

#### Pathways and functional enrichment analysis

The Ingenuity Pathway Analysis (IPA 7.0) was used to identify the pathways and biological functions affected by proteins that are specifically associated with HCC or HCC and Cirrhosis (CIR). The knowledge base of this software consists of functions, pathways and network models derived by systematically exploring the peer reviewed scientific literature. A detailed description of IPA analysis is available at the Ingenuity Systems’ web site (http//http://www.ingenuity.com). This software calculates a P-value for each pathway according to the fit of user’s data to IPA database by the one-tailed Fisher exact test. Pathways with multiple test corrected P-values <0.05 were considered significantly affected.

#### Interactive network analysis

To gain further molecular insight HCC progression, we performed systems biology oriented analysis on proteins that are HCC specific or disease specific (HCC + CIR) using Ingenuity Pathway Analysis (IPA) 7.0. The networks were developed on the basis of protein-protein; protein-DNA, protein-RNA and protein-chemical interactions obtained public databases and experimentally validated literature. The significance of the effect on the network was determined on the basis of score derived from the P value of the one-tailed Fisher exact test [Score = −log (P value)] and indicates the likelihood of focus proteins appearing together in the network due to random chance. A score of 2 or higher has at least a 99% probability of not being generated by random chance alone. The ability to rank the networks based on their relevance to the queried data sets allows for prioritization of networks with the highest impact on a disease process. The key focus hubs in the network were identified using degree of connectivity (number of interactions for a node with other network proteins). The focus hubs are likely critical for overall function of the network and, thus, interruption of such proteins by therapeutic intervention is anticipated to perturb the whole network of proteins.

### Target validation

Target validation of proteomic results was performed on 18 independent tissue samples (9 AA, and 9 CA). Three differentially expressed proteins were identified and selected based on unsupervised hierarchical clustering and the Interactive Network Analysis. We validated the expression of these proteins using quantitative real-time RT-PCR (qRT-PCR) or Western blotting (WB).

#### Quantitative real-time PCR (qRT-PCR)

Total RNA was extracted from tissue homogenates using the RNeasy mini kit (Qiagen, Valencia, CA) and quantified using Nanodrop spectrophotometry (ThermoScientific, Wilmington, DE). RNA quality was assessed with the use of a Bioanalyzer 2100 (Agilent Technologies, Santa Clara, CA). One microgram of RNA was reverse transcribed to complementary DNA (cDNA) using Superscript II in accordance with manufacturer’s instructions (Invitrogen). qRT-PCR was run in technical duplicates for each reaction using 50 ng cDNA from at least triplicate of normal, HCV+/HCC- and HCV+/HCC + samples of AA and CA. The validated genes were: Serotransferrin (TF), Filamin A Alpha (FLNA), Apolipoprotein A-1 (APOA-1), and hepatocyte nuclear factor 4α (HNF4α). Relevant information on RT-PCR primers used to detect the expression of these genes is shown in Table 1, qRT-PCR data for each sample were normalized using 18S rRNA gene. Data were collected using the ABI PRISM 7500 sequence detection system (Applied Biosystems, Forster City, CA). Graphs were prepared from normalized data relative to 18S rRNA and fold changes were calculated using the ddCt method as, previously described [28] and detailed by Applied Biosystems (http://www.appliedbiosystems.com). Statistical analysis of these data was performed with a two-sided *t*-test or with a two-sided Wilcoxon rank-sum test if the expression data not follow normal distribution.

#### Western blot (WB) analysis

Selected protein expression in tissue samples was verified by WB analysis, as previously reported [28]. Briefly, 20 μg of total protein were separated by SDS-PAGE (12% polyacrylamide gel) and transferred onto nitrocellulose membrane (Millipore, Bradford, MA). TF, APOA1 and HNF4α proteins were identified using mouse anti-transferrin, mouse anti-APOA-1 (Santa Cruz Biotechnology), and rabbit anti-HNF4α (Epitomics) primary antibodies. GAPDH (Rockland) was used as a loading control. Protein expression was visualized after incubation with secondary anti-mouse or anti-rabbit antibodies conjugated with horseradish peroxidase and enhanced chemiluminescence reagent (Thermo Scientific, Rockford, IL). Immunoblots were developed on a ChemiDoc XRS gel Imaging System (Bio-Rad Laboratories, Hercules, CA) for immunodetection. The intensity of protein staining was determined with the ChemiDoc Imager using Quantity One Software (Bio-Rad Laboratories, Hercules, CA).

#### Statistical analysis

The data were expressed as mean±SD, and analyzed with the Student’s *t*-test between two groups. Changes were considered statistically significant if the *P*-value was <0.05.

## Results

### Clinical characteristics of the study population

A total of 26 liver and tumor tissue samples from CA and AA populations were used in this study (Additional file 2: Table S1). There were no significant differences of age and sex between cases in the two groups. In addition, the cirrhotic cases (HCV+/HCC-) of the AA group had statistically different laboratory results for aspartate aminotransferase (AST), and alanine aminotransferase (ALT) (p < 0.05) compared to CA group. There were no significance differences of the laboratory values for albumin, total albumin and hemoglobin between cases in the two groups.

### Clustering analysis of identified proteins can discriminate between normal and diseased stage

**Figure 1:**
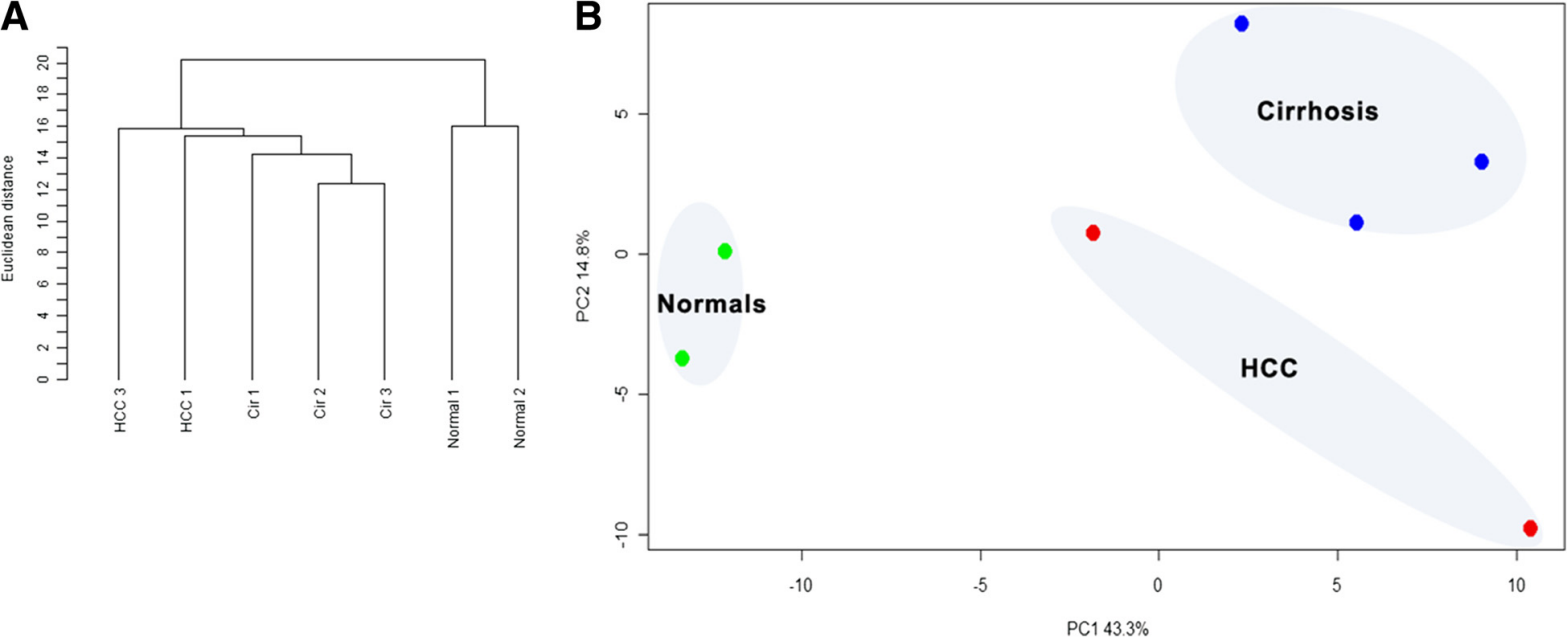
**Unsupervised analysis of normalized proteomics data obtained from Normal**, **Cirrhosis (Cir) and HCC subjects: A) Unsupervised Clustering B) Principal component analysis.** Unsupervised Pearson Correlation based cluster of Normal, Cirrhosis (Cir) and HCC subjects depicts two major clusters containing Normal’s and Diseased (Cir, HCC) subjects. B) The first principal component with highest variation (43%) is shown on the X-axis and separates the samples on the basis of Diseased vs Normal status. The second component with median variance (15%) is displayed on the Y-axis and separates the sample on the basis of the disease progression (Cirrhosis vs. HCC). The Normal, Cirrhosis and HCC samples formed three separate clusters on PCA plot.

Using the results from Protein Pilot 3.0, we identified a total of 238 proteins with at least 1 peptide of >95% confidence. The normal, CIR, and HCC formed separate clusters on the PCA plot (Figure 1B) and we determined that samples separated on the basis of disease status (e.g. HCC, CIR vs. Normal) along primary component (PC1) accounted for 43.3% of the variation between samples. We also performed hierarchical clustering using a euclidean distance metric (Figure 1A), and demonstrated two major clusters linked to disease and normal samples. In the diseased cluster, subclusters depicting significant similarity within cirrhosis and HCC samples were observed.

### Identification of differentially expressed proteins between normal and diseased states in CA population

**Figure 2:**
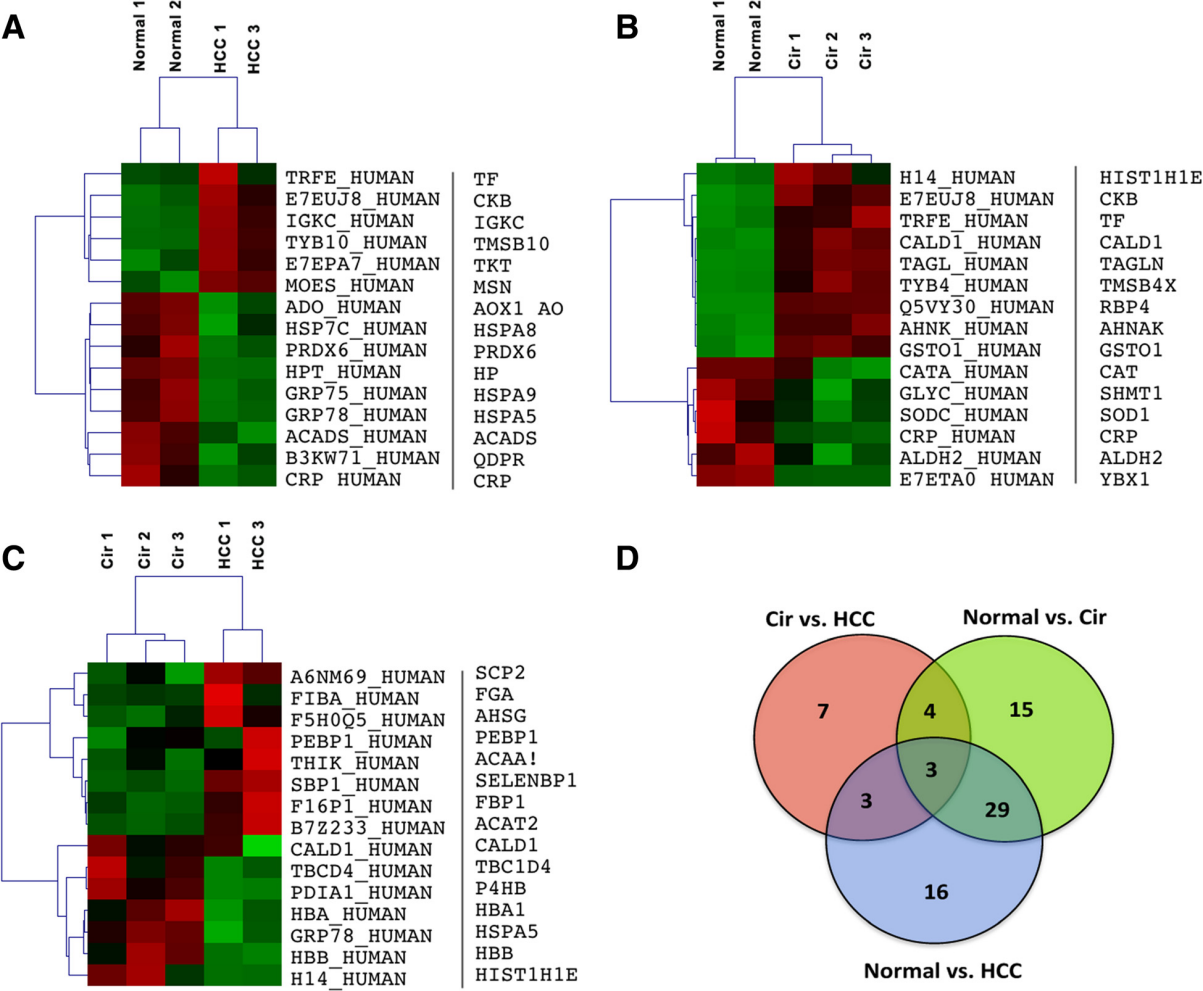
**Identification and comparison of differentially expressed proteins (DEP) identified from different supervised analysis.** Heat maps of fifteen differentially expressed genes that were selected by following supervised analysis **A)** Normal vs. Cirrhosis, **B)** Normal vs. HCC, and **C)** Cirrhosis vs. HCC. The columns represent the samples and the rows represent the proteins. Protein expression is depicted with a pseudocolor scale (−2 to 2); red denoting high expression level and green denoting low expression level. **D)** Venn Diagram comparing the significantly differentially expressed proteins identified from following comparisons i) Normal vs. Cirrhosis, ii) Normal vs. HCC, and iii) Cirrhosis vs. HCC.

**Figure 3:**
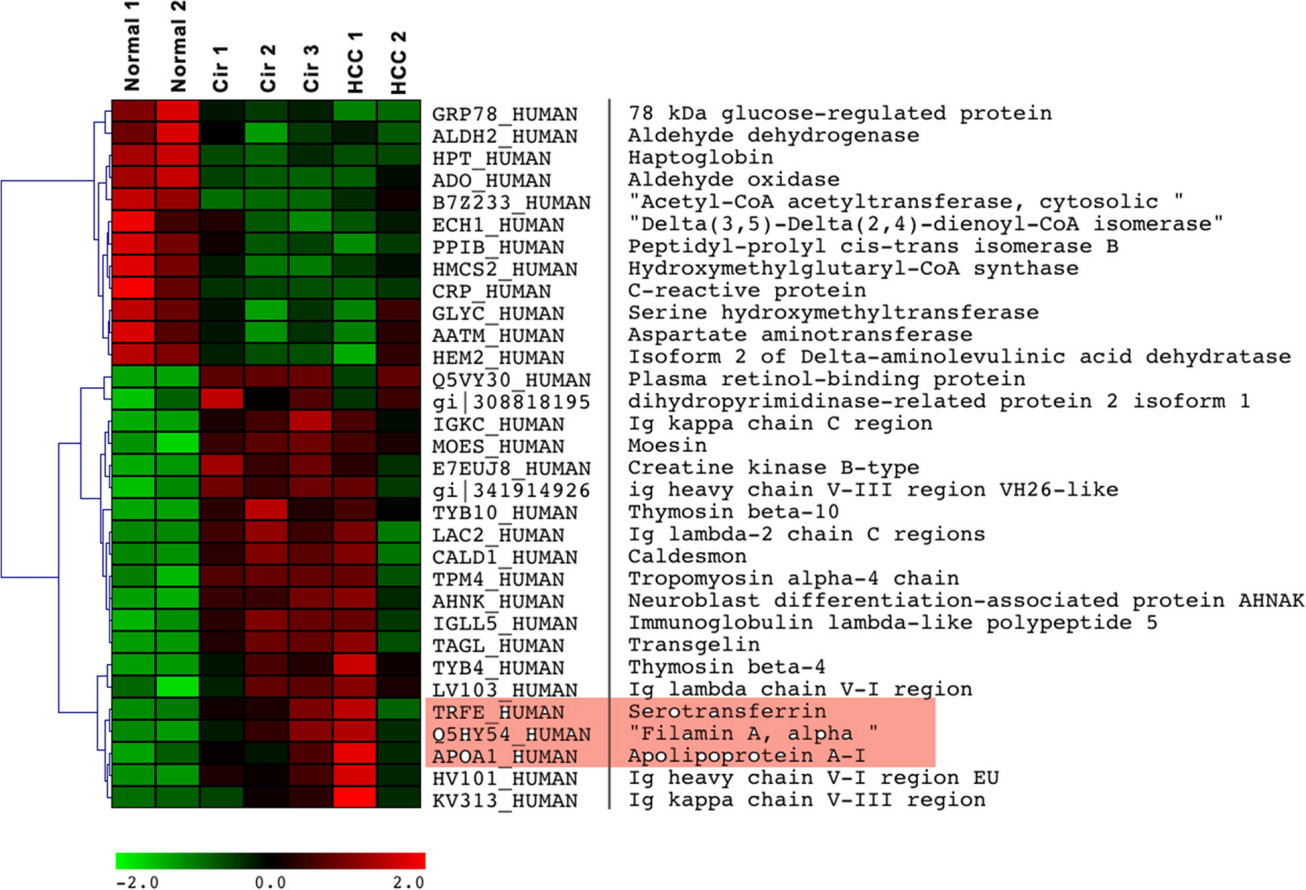
**Heat map of thirty-two proteins differentially expressed in both Cirrhosis and HCC as compared to Normal.** These proteins represent a potential signature depicting progression of disease. The columns represent the samples and the rows represent the proteins. Protein expression is shown with a pseudocolor scale (−2 to 2); red denoting high expression level and green denoting low expression level. Proteins validated using qRT-PCR or immunoblotting are highlighted in the heat map.

To identify the differentially expressed proteins (DEP), the relative protein expression values were compared between groups (Normal vs. Cirrhosis (CIR), Normal vs. HCC, Cirrhosis vs. HCC). The identification of proteins differentially expressed in cirrhotic and HCC patient groups relative to the normal group were of interest as these could provide leads for potentially useful diagnostic and prognostic biomarkers for disease progression. Thus, Figure 2 shows heat maps of fifteen differentially expressed proteins that were selected by this supervised analysis, as outlined in the Bioinformatics analysis section. For example, Figure 2A shows differentially expressed proteins of HCC group compared to normal; six of which were overexpressed in HCC compared to nine proteins over-expressed in normal tissues. Similarly, a comparison between the cirrhotic groups versus the normal group identified nine proteins overexpressed in cirrhotic group versus six that were over-expressed in the normal group (Figure 2B). As shown in Figure 2D, thirty-two proteins (about 15% of proteins identified) overlapped between HCC and cirrhotic groups as compared to the normal. These thirty-two proteins met our definition for differential expression (see Experimental Procedures) in comparison between CIR, HCC to the normal group. Thus, Table 2 and Figure 3 show the thirty-two differentially expressed proteins (DEP): twenty were overexpressed (iTRAQ ratios of ≥2.0) and twelve were under-expressed (iTRAQ ratios ≤0.5).

A literature search showed that all DEP have previously been associated with hepatitis and HCC as a result of HCV infection. For example, high plasma retinol-binding protein (RBP4) has been reported to be associated with the pathogenesis of insulin resistance in type 2 diabetes [29]. RBP4 was also shown to have prognostic significance as a marker in patients with chronic liver disease and cirrhosis-induced by genotype 1 HCV infection [30]. Similarly, transgelin (TAGL) has been shown to be associated with cell migration and invasion of cancer stem cells [31]. TAGL was also shown to have a potential prognostic significance in HCC [32].

### Gene ontology annotation (GO analysis)

**Figure 4:**
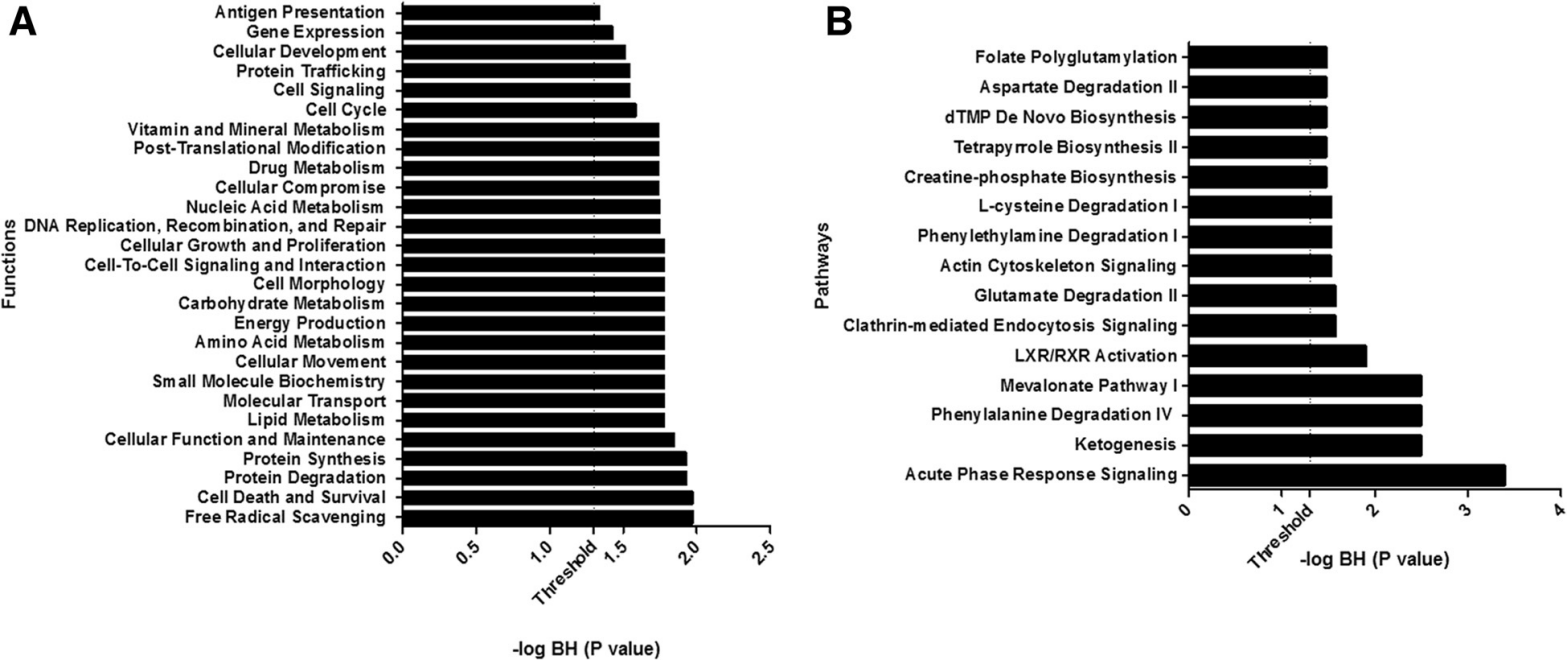
**Pathways and functional enrichment analysis of proteins differentially expressed both in Cirrhosis and HCC as compared to Normal A) Functional enrichment analysis B) Pathways enrichment analysis.** For Figure 4A or Figure 4B each bar represents a significantly enriched pathway or function as determined using the multiple test corrected Fisher’s Exact Test P-value. The P-value is depicted as –log10 (BH P value) on primary X-axis. The analysis for canonical pathways and functions was performed using Ingenuity Systems interactions.

We subjected the differentially expressed proteins to GO analysis and categorized them according to molecular function, biological processes and pathways. When we analyzed these proteins for molecular function (Figure 4A), we found that over 50% of proteins (P < 10^-1.55^) were grouped under “hepatic metabolism” such as vitamin and mineral metabolism, drug metabolism, nucleic acid metabolism, carbohydrate metabolism, amino acid metabolism and lipid metabolism (Figure 4A). The remaining differentially expressed proteins were grouped under “stress-related process” (P < 10^-2.0^), “protein-related processes” (P < 10^-1.9^), and “cell signaling” (P < 10^-1.8^).

We also grouped the differentially expressed proteins into molecular pathways (Figure 4B). We found that a significant number of proteins were grouped under various canonical pathways. However, the major pathway identified is “acute phase response signaling” (P < 10^-3.5^). This is a rapid inflammatory response that provides protection against various types of infection including viral infection such as HCV. As a consequence of this acute phase response most of identified proteins were thus included under “hepatic metabolism” as shown in Figure 4A.

### Differentially expressed proteins are involved in a number of pathways associated with disease progression

**Figure 5:**
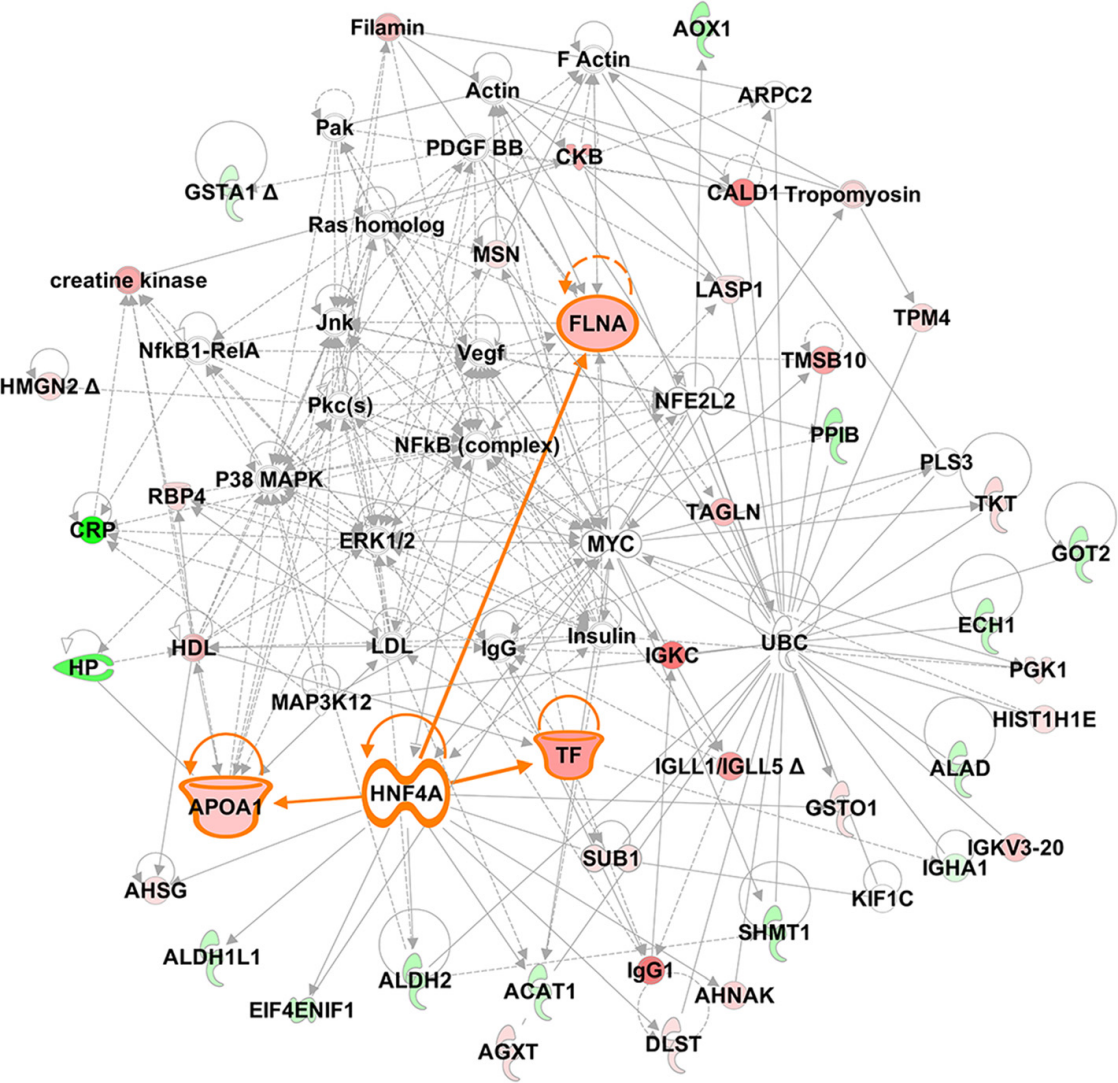
**Interactive Network representation of the cellular functions and pathways affected by thirty-two proteins that are commonly altered in Cirrhosis and HCC as compared to Normal.** The network is enriched with proteins significantly linked to cell movement, connectivity tissue disorder and cancer. We used the Ingenuity Pathways Analysis tool (http://www.ingenuity.com) to generate the networks of proteins that are only differentially expressed in Cirrhosis and HCC as compared to normal. The intensity of the node color indicates the degree of up-regulation (red), down-regulation (green) or no effect (white) in HCC as compared to Normal samples.

We performed Interactive Network analysis on the differentially expressed proteins using the Ingenuity Pathway Analysis (IPA) tool. As shown in Figure 5, the network consisted of a cluster of seventy proteins, our thirty-two DEP (Table 2), and thirty-eight additional proteins. The network is enriched with proteins significantly linked to cell movement, connectivity tissue disorder and cancer. This network also exhibited focus hubs containing NFκB, ERK1/2, UBC, p38MAPK, and HNF4α, all which regulate inflammation, and survival and proliferation of tumor cells. The majority of the molecular targets identified in this study (Table 2 and Figure 3) were, in fact, regulated by these focus hubs. As shown in Figure 5, there is a high degree of interaction between HNF4α (a focus hub) and target genes such as serotransferrin (TF) and apolipoprotein lipase A1 (APOA1), and to a lesser degree with filamin-A alpha (FLNAα) (labeled orange). These data suggest that interruption of these pathways may provide a means to the development of molecularly targeted therapies for HCV-induced HCC [33]. Thus, the expression levels of HNF4α (a focus hub) and interacted proteins (TF, APOA1, and FLNA) were selected for further validation using qRT-PCR and/or immunoblotting.

### Target validation

**Figure 6:**
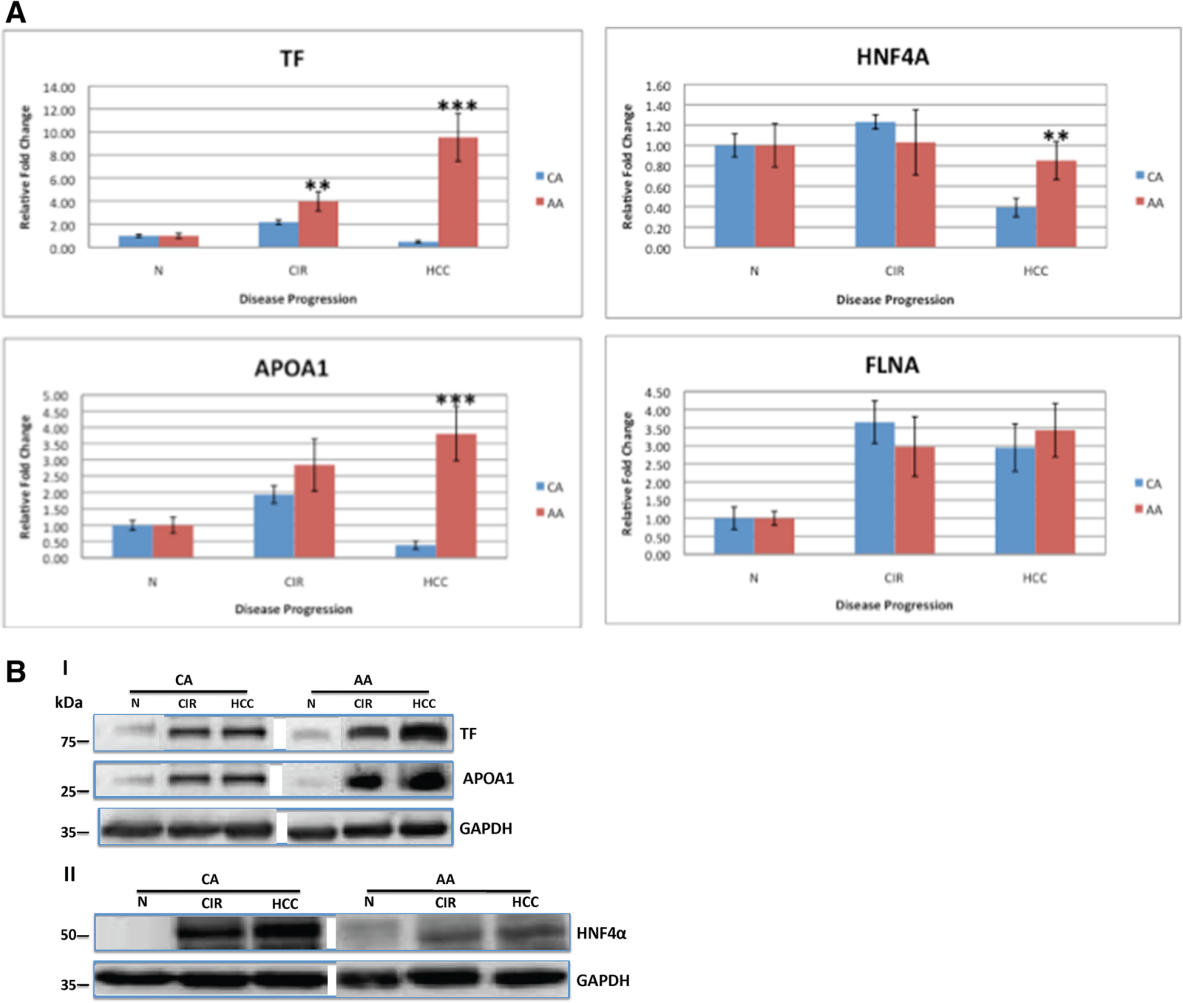
**Target validation of DEP in tissue samples of CA and AA. A)** Real-time qRT-PCR, **B)** A representative of Western blotting analysis. **A)** Real-time qRT-PCR detected the relative mRNA expression levels of transferrin (TF), apolipoprotein A1 (APOA1), hepatocyte nuclear factor4α (HNF4α) and filamin A (FLNA). 18S rRNA was used as the normalization standard. Compared to CA, AA tissues had an obvious up-regulation of TF in CIR and HCC samples (p < 0.05 and 0.001, respectively) and up-regulation of APOA1, and HNF4α in HCC samples (p < 0.001; p < 0.05, respectively). Bars = means ± SD. There was no significant difference in the expression of FLNA between the two groups. **B)** A representative of immunoblot analysis result of (I) TF and APOA1, (II) HNF4α in tissue samples of AA and CA. GAPDH was used as a loading control. Compared to CA, AA tissue samples (CIR, HCC) show an obvious increase in protein levels of TF and APOA1, but decreased protein levels of HNF4α.

We were able to confirm the differential expression of TF, APOA1, FLNA, and HNF4α by qRT-PCR or WB analyses using independent sets of 18 tissue samples (9 AA, 9 CA; 3 tissues/group). These four proteins were selected for validation based on their expression using both hierarchal clustering analysis (Figure 3) and Interactive Network analysis (Figure 5). Figure 6A shows the relative mRNA expression levels of TF, APOA1, FLNA and HNF4α as normalized to 18S rRNA in AA (red) & CA (blue) tissue samples. The mRNA expression levels of TF were 2 fold (p < 0.05) and 18 fold (p < 0.001) higher in Cir & HCC tissues of AA samples compared to CA. Similarly, the mRNA expression levels of APOA1 and HNF4α were 7 fold (p < 0.001) and 2 fold (p < 0.05) higher, respectively, in HCC of AA samples compared to CA. No significant changes in FLNA mRNA expression levels were observed. A similar trent was noticed at the protein using WB. Figure 6B shows representative immunoblots of TF and APOA1 expression in normal (N), Cir (cirrhosis), and HCC in both AA and CA protein extracts with GAPDH employed as a loading control. Compared with normal tissues, Cir and HCC tissues have a detectable increase in the steady-state levels of TF and APOA1 in AA as compared to CA. In contrast however, the expression levels of HNF4α protein were different when compared to the mRNA levels seen in Figure 6A. Figure 6B shows clearly that the steady-state levels of HNF4α protein are reduced in AA samples (Cir and HCC) as compared to CA.

## Discussion

Cancer disparities in incidence and death rates exist among various racial and ethnic groups. These disparities are clearly documented in many aggressive human cancers (e.g., breast, colon, ovarian, prostate and bladder) [34-38]. There have been several studies suggesting that this phenomenon is potentially caused by a multitude of factors, including social and cultural experience, shared behaviors, environmental exposure and variations in genetic background. Environmental factors have been identified as risk factors for cancers, and these can affect cancer disparities between races and ethnicities. For example, persistent infection with HCV is a well-documented risk factor for HCC. There are clear racial/ethnic disparities in disease prevalence, treatment and outcome to make it a particularly important health problem in minorities [1,9]. While much of the existing literature has focused on noting the presence of disparities in HCV-induced HCC, little is known about specific biological pathway differences within the context of racial background.

In this study, we hypothesized that HCV-induced oxidative stress activates sets of host-specific genes (molecular signatures) that are associated with the disease state and are ethnically/racially distinct. These sets of genes could confer various biological properties responsible for the observed disparities. Identification of these molecular signatures could provide us with valuable insights into the biological factors (gene expression, protein activity) that contribute to HCV-induced HCC health disparities.

We performed relative quantitative proteomic profiling to identify differential protein expression between HCV-induced cirrhosis (CIR) and HCV-induced hepatocellular carcinoma (HCC) directly compared to normal in tissue samples obtained from Caucasian American (CA) patients and cross-validated protein expression on tissue samples obtained from African American (AA) patients. We identified thirty-two proteins that were significantly differentially expressed in CIR and HCC compared to normal liver tissue samples of CA (Table 2 and Figure 3). Interestingly, a significant number of these proteins had previously been reported to be involved in HCV/HCC disease progression For example, proteins identified in this study like moesin (MSN) (Figure 2A), retinol-binding protein (RBP4), and transgelin (TAGL) (Figure 2B) have been involved in viral induced HCC. MSN was shown to be involved in viral related invasion and metastasis of HCC [39]. RBP4 was demonstrated to have a prognostic significance as a marker in patients with chronic liver disease and cirrhosis-induced by genotype 1 HCV infection [30]. Similarly, TAGL was shown to possess a potential prognostic significance in HCC [32].

We used Ingenuity Pathway Analysis (IPA) to assess disease and functions/pathways association of differentially expressed proteins (DEP). Top associated network functions for DEP (Figure 4A) were: 1) free radical scavenging 2) cell death and survival 3) protein degradation 4) protein synthesis 5) cellular function and maintenance 6) lipid metabolism, and 7) molecular transport. As shown in Figure 3, we identified many proteins that are involved in many of these cellular functions such as stabilization of actin filament structure. These include Filamin A (FLNA), Moesin (MOES), Caldesmon (CALD1) and tropomyosin alpha-4 (TYB4). These proteins have been shown to be involved in cellular migration, invasion and metastasis of HCC [40-42]. Other proteins such as Apolipoprotein A1 (APOA1), and Serotransferrin (TF) that are involved in lipid metabolism and molecular transport were identified as DEP. Both of these proteins have been shown to be associated with HCV-induced HCC [43-45].

In this study, major canonical biological pathways identified are: 1) acute phase response signaling 2) ketogenesis 3) phenylalanine degradation IV 4) mevalonate pathway I, and 5) LXR/RXR activation. These pathways are known be associated with HCV-induced HCC. For example, the acute phase response is a rapid inflammatory response that provides protection against the viral infection using innate defense mechanisms [46]. The majority of the DEP are involved in the acute phase signaling pathway and thus strongly implicated in HCV infection.

Another major pathway that was identified is the liver X receptor (LXR)/retinoid X receptor (RXR) activation. LXR is a key player in the control of numerous metabolic pathways and along with RXR, LXR plays a crucial role in linking bile acid with lipoprotein, lipid and glucose metabolism (hepatic lipogenesis). LXR has been shown as a major contributor to HCV-induced steatosis and in the efficient replication of HCV [47,48].

The Network Analysis has identified many focus hubs (e.g., NFκB, ERK1/2, UBC, and p38MAPK) with high degree of interactions. These focus hubs are involved in the overall pathophysiological response to HCV infection [49,50]. It is known that HCV infection enhances the generation of reactive oxygen species (ROS) that act through these hub molecules [51]. This process has been suggested as one of the mechanisms for HCV induced hepatic fibrosis.

The Network Analysis has also identified a high degree of interaction between hepatocyte nuclear factor (HNF4α) (focus hub) and target proteins such TF and APOA1, and to a lesser degree with FLNA (Figure 5). HNF4α, a highly conserved member of the nuclear receptor (NR) superfamily of ligand-dependent transcription factors, is known as a master regulator of liver-specific gene expression [52], especially those genes involved in lipid transport such as APOA1 [53], glucose metabolism and iron transport, such as transferrin (TF) [54,55]. Therefore, the expression of TF, APOA1, FLNA and HNF4α was selected for further validation in CA and AA tissue samples using q-RT-PCR or WB.

It is very clear in Figure 6A &6B that the expression levels of TF and APOA1 are higher in AA compared to CA tissue samples. It is known that AA patients with chronic HCV have elevated levels of serum markers of iron stores and altered cholesterol and triglyceride levels [56,57]. Hence, the levels of both markers are elevated in AA samples. The expression of both TF and APOA1 is known to be regulated by the transcription factor HNF4α [33]. Nevertheless, the levels of HNF4α protein itself are reduced in AA compared to CA tissue samples, as shown in Figure 6B. This differential dysregulation of HNF4α expression in Figures 6A &6B has been shown by Sladek’s group in colon cancer [58]. It is not clear why the levels of HNF4α are altered in AA versus CA tissue samples. There are many factors that could alter the expression and function of HNF4α like single nucleotide polymorphisms (SNPs), diet, stress response, severity of disease, and regulatory molecules like transcription factors, co-regulators, and miRNAs [52]. Recent studies showed that alteration of HNF4α protein expression could provoke the initiation of HCC [59,60]. Thus, it is conceivably possible that racial disparities in HCC could be a consequence of differential dysregulation of HNF4α expression in AA patients. Further study using larger clinical samples size is warranted to confirm this observation.

In conclusion, through the use of comparative proteomic analysis by relative quantitation with isobaric tagging, we identified differentially expressed proteins that can distinguish between HCV-induced cirrhotic liver and HCV-induced hepatocellular carcinoma. Many of these proteins are involved in biological pathways pertinent to the overall pathophysiological response to HCV infection. Target validation analyses showed that some of these proteins are highly expressed in AA tissue samples compared to CA. In contrast, our study also indicated that there is a differential dysregulation of HNF4α expression in AA compared to CA. Alteration in HNF4α levels could be one of the reasons for the observed racial disparities in HCC seen between both groups. Further validation of these markers in a larger study would greatly improve our understanding of the molecular mechanisms behind this racial disparity in HCC.

## Abbreviations

HCV: Hepatitis C virus; HCC: Hepatocellular carcinoma; iTRAQ: Isobaric labeling tags for relative and absolute quantitation; CIR: Cirrhosis; CA: Caucasian Americans; AA: African Americans; TF: Transferrin; APOA1: Apolipoprotein A1; HNF4α: Hepatocyte nuclear factor4α; FLNA: Filamin A; qRT-PCR: Quantitative real-time RT-PCR; WB: Western blotting; PCA: Principal component analysis; IPA: Ingenuity pathway analysis; DEP: Differentially expressed proteins; NR: Nuclear receptor.

## Competing interests

The authors declare that they have no competing interest.

## Authors’ contributions

Conceived and designed the experiments: SSD. Performed the experiments: STD XSF. Analyzed the data: MKB XSF. Contributed reagents/materials/analysis tools: SSD MKB DK. Wrote the paper: STD MKB DK SSD. All authors read and approved the final manuscript.

## Supplementary Material

Additional file 1 Peptide MS Spectra Data. Click here for file

Additional file 2: Table S1 Clinical characteristics of the study population. Click here for file

## References

[B1] El-Serag HB Hepatocellular carcinoma: current concepts N Engl J Med 2011 365 1118 1127 10.1056/NEJMra1001683 21992124

[B2] Edlin BR Perspective: test and treat this silent killer Nature 2011 474 S18 S19 21613999 10.1038/474S18a PMC4751873

[B3] Jacobson IM Davis GL El-Serag H Negro F Trépo C Prevalence and challenges of liver diseases in patients with chronic hepatitis C virus infection Clin Gastroenterol Hepatol 2010 8 924 933 10.1016/j.cgh.2010.06.032 20713178

[B4] Castello G Scala S Palmieri G Curley SA Izzo F HCV-related hepatocellular carcinoma: From chronic inflammation to cancer Clin Immunol 2010 134 237 250 10.1016/j.clim.2009.10.007 19910258

[B5] Caldwell S Park SH The epidemiology of hepatocellular cancer: From the perspective of public health problem to tumor biology J Gastroenterol 2009 44 96 101 10.1007/s00535-008-2258-6 19148801

[B6] Mathur AK Heimbach J Steffick DE Sonnenday CJ Goodrich NP Merion RM Donation after cardiac death liver transplantation: predictors of outcome Am J Transplant 2010 10 2512 2519 10.1111/j.1600-6143.2010.03293.x 20977642

[B7] Trooskin SB Navarro VJ Winn RJ Axelrod DJ McNeal AS Velez M Herrine SK Rossi S Hepatitis C risk assessment, testing and referral for treatment in urban primary care: role of race and ethnicity World J Gastroenterol 2007 13 1074 1078 17373742 10.3748/wjg.v13.i7.1074 PMC4146870

[B8] Satapathy SK Lingisetty CS Proper S Chaudhari S Williams S Equally poor outcomes to pegylated interferon-based therapy in African Americans and Hispanics with chronic hepatitis C infection J Clin Gastroenterol 2010 44 140 145 10.1097/MCG.0b013e3181ba9992 19826275

[B9] Artinyan A Mailey B Sanchez-Luege N Khalili J Sun CL Bhatia S Wagman LD Nissen N Colquhoun SD Kim J Race, ethnicity, and socioeconomic status influence the survival of patients with hepatocellular carcinoma in the United States Cancer 2010 116 1367 1377 10.1002/cncr.24817 20101732

[B10] Gebo KA Chander G Jenckes MW Ghanem KG Herlong HF Torbenson MS El-Kamary SS Bass EB Screening tests for hepatocellular carcinoma in patients with chronic hepatitis C: a systematic review Hepatology 2002 36 S84 S92 12407580 10.1053/jhep.2002.36817

[B11] Bertino G Ardiri A Malaguarnera M Malaguarnera G Bertino N Calvagno GS Hepatocellular carcinoma serum markers Semin Oncol 2012 2012 39 410 433 22846859 10.1053/j.seminoncol.2012.05.001

[B12] Paul SB Gulati MS Sreenivas V Madan K Gupta AK Mukhopadhyay S Acharya SK Evaluating patients with cirrhosis for helpatocellular carcinoma: value of clinical symptomatology, imaging and alpha-fetoprotein Oncology 2007 72 117 123 10.1159/000111717 18087192

[B13] Richardson P Duan Z Kramer J Davila JA Tyson GL El-Serag HB Determinants of serum alpha-fetoprotein levels in hepatitis C-infected patients Clin Gastroenterol Hepatol 2012 10 428 433 10.1016/j.cgh.2011.11.025 22155556 PMC3311729

[B14] Nam SW Park JY Ramasamy A Shevade S Islam A Long PM Park CK Park SE Kim SY Lee SH Park WS Yoo NJ Liu ET Miller LD Lee JY Molecular changes from dysplastic nodule to hepatocellular carcinoma through gene expression profiling Hepatology 2005 42 809 818 10.1002/hep.20878 16175600

[B15] Wang W Peng JX Yang JQ Yang LY Identification of gene expression profiling in hepatocellular carcinoma using cDNA microarrays Dig Dis Sci 2009 54 2729 2735 10.1007/s10620-008-0667-2 19117127

[B16] Gehrau RC Archer KJ Mas VR Maluf DG Molecular profiles of HCV cirrhotic tissues derived a panel of markers with clinical utility for hepatocellular carcinoma surveillance PLoS One 2012 7 e40275 10.1371/journal.pone.0040275 22792259 PMC3390353

[B17] Chen G Gharib TG Huang CC Taylor JM Misek DE Kardia SL Giordano TJ Lannettoni MD Orringer MB Hanash SM Beer DG Discordant protein and mRNA expression in lung adenocarcinomas Mol Cell Proteomics 2002 1 304 313 10.1074/mcp.M200008-MCP200 12096112

[B18] Uto H Kanmura S Takami Y Tsubouchi H Clinical proteomics for liver disease: a promising approach for discovery of novel biomarkers Proc Natl Acad Sci U S A 2010 8 70 80 10.1186/1477-5956-8-70 PMC3023778 21192835

[B19] Mas VR Maluf DG Archer KJ Yanek K Bornstein K Fisher RA Proteomic analysis of HCV cirrhosis and HCV-induced HCC: Identifying biomarkers for monitoring HCV-cirrhotic patients awaiting liver transplantation Transplantation 2009 87 143 152 10.1097/TP.0b013e318191c68d 19136905 PMC2699556

[B20] Nomura F Sogawa K Noda K Seimiya M Matsushita K Miyazaki M Yokosuka O Serum anti-Ku86 is a potential biomarker for early detection of hepatitis C virus-related hepatocellular carcinoma Biochem Biophys Res Commun 2012 421 837 843 10.1016/j.bbrc.2012.04.099 22554520

[B21] Diamond DL Jacobs JM Paeper B Proll SC Gritsenko MA Carithers RJ Jr Larson AM Yeh MM 2nd Camp DG Smith RD Katze MG Proteomic profiling of human liver biopsies: Hepatitis C virus-induced fibrosis and mitochondrial dysfunction Hepatology 2007 46 649 657 10.1002/hep.21751 17654742

[B22] Diamond DL Krasnoselsky AL Burnum KE Monroe ME Webb-Robertson B-J McDermott JE Yeh MM Dzib JF Susnow N Storm S Proll SC Bellisle SE Purdy DE Rasmussen AL Walters KA Jacobs JM Gritsenko MA Camp DG Bhattacharya R Perkins JD Carithers RL Jr Liou IW Larson AM Benecke A Waters KM Smith RD Katze MG Proteome and computational analyses reveal new insights into the mechanisms of hepatitis C virus-mediated liver disease posttransplantation Hepatology 2012 56 28 38 10.1002/hep.25649 22331615 PMC3387320

[B23] Zhao S Zun M Chu Y Zhu T Wang Y Yan L Xun X Song J Shao M Proteome analysis identifies the role of heat stress in production of progeny HCV in Huh7 cells harboring intact HCV Intervirology 2008 51 196 202 10.1159/000151631 18753793

[B24] Sato S Fukasawa M Yamakawa Y Natsume T Suzuki T Shoji I Alzaki H Miyamura T Nishijima M Proteomic profiling and lipid droplet proteins in Hepatoma cell lines expressing hepatitis C virus core protein J Biochem 2006 139 921 930 10.1093/jb/mvj104 16751600

[B25] McCully JD Bhasin MK Daly C Guerrero MC Dillon S Liberman TA Cowan DB Mably JD McGowan FX Levitsky S Transcriptomic and proteomic analysis of global ischemia and cardioprotection in the rabbit heart Physiol Genomics 2009 38 125 137 10.1152/physiolgenomics.00033.2009 19454556 PMC2712218

[B26] Glen A Evans CA Gan CS Cross SS Hamdy FC Gibbins J Lippitt J Eaton CL Noirel J Wright PC Rehman I Eight-plex iTRAQ analysis of variant metastatic human prostate cancer cells identifies candidate biomarkers of progression: An exploratory study Prostate 2010 70 1313 1332 20623638 10.1002/pros.21167

[B27] Shilov IV Seymour SL Patel AA Loboda A Tang WH Keating SP Hunter CL Nuwaysir LM Schaeffer DA The Paragon Algorithm, a next generation search engine that uses sequence temperature values and feature probabilities to identify peptides from tandem mass spectra Mol Cell Proteomics 2007 6 1638 1655 10.1074/mcp.T600050-MCP200 17533153

[B28] Daoud SS Munson PJ Reinhold W Young L Prabhu VV Yu Q LaRose J Kohn KW Weinstein JN Pommier Y Impact of p53 knockout and topotecan treatment on gene expression profiles in human colon carcinoma cells: A pharmacogenomic study Cancer Res 2003 63 2782 2793 12782583

[B29] Yang Q Graham TE Mody N Preitner F Peroni OD Zabolotny JM Kotani K Quadro L Kahn BB Serum retinol binding, protein 4 contributes to insulin resistance in obesity and type 2 diabetes Nature 2005 436 356 362 10.1038/nature03711 16034410

[B30] Petta S Cammà C Di Marco V Alessi N Barbaria F Cabibi D Cabibi D Caldarella R Ciminnisi S Licata A Massenti MF Mazzola A Tarantino G Marchesini G Crazi A Retinol-binding protein 4: a new marker of virus-induced steatosis in patients infected with hepatitis C virus genotype 1 Hepatology 2008 48 28 37 18506842 10.1002/hep.22316

[B31] Lee EK Han GY Park HW Song YJ Kim CW Transgelin promotes migration and invasion of cancer stem cells J Proteome Res 2010 9 5108 5117 10.1021/pr100378z 20707403

[B32] Chen XL Zhou L Yang J Shen FK Zhao SP Wang YL Hepatocellular carcinoma-associated protein markers investigated by MALDI-TOF MS Mol Med Rep 2010 3 589 596 21472284 10.3892/mmr_00000302

[B33] Mogilenko DA Dizhe EB Shavva VS Lapikov IA Orlov SV Lapikov IA Orlov SV Perevozchikov AP **Role of the nuclear receptors HNF4**α, **PPAR**α, **and LXRs in the TNF**α-**mediated inhibition of human apolipoprotein AI gene expression in HepG2 cells** Biochemistry 2009 48 11950 11960 10.1021/bi9015742 19883121

[B34] DeSantis C Siegel R Bandi P Jemal A Breast cancer statistics, 2011 CA Cancer J Clin 2011 61 409 418 21969133 10.3322/caac.20134

[B35] Dimou A Syrigos KN Saif MW Disparities in colorectal cancer in African-Americans vs Whites: before and after diagnosis World J Gastroenterol 2009 15 3734 3743 10.3748/wjg.15.3734 19673013 PMC2726450

[B36] Bristow RE Zahurak ML Ibeau OA Racial disparities in ovarian cancer surgical care: a population-based analysis Gynecol Oncol 2011 121 364 368 10.1016/j.ygyno.2010.12.347 21288564

[B37] Chornokur G Dalton K Borysova ME Kumar NB Disparities at presentation, diagnosis, treatment, and survival in African American men, affected by prostate cancer Prostate 2011 71 985 997 10.1002/pros.21314 21541975 PMC3083484

[B38] Jacobs BL Montgomery JS Zhang Y Skolarus TA Weizer AZ Hollenbeck BK Disparities in bladder cancer Urol Oncol 2011 30 81 88 22127016 10.1016/j.urolonc.2011.08.011

[B39] Lara-Pezzi E Serrador JM Montoya MC Zamora D Yáñez-Mó M Carretero M Furthmayr H Sanchez-Madrid F Lopez-Cabrera M The hepatitis B virus X protein (HBx) induces a migratory phenotype in a CD44-dependent manner: possible role of HBx in invasion and metastasis Hepatology 2001 33 1270 1281 10.1053/jhep.2001.1270 11343256

[B40] Sasaki Y Yamamura H Kawakami Y Yamada T Hiratsuka M Kameyama M Ohigashi H Ishikawa O Imaoka S Ishiguro S Takahashi K Expression of smooth muscle calponin in tumor vessels of human hepatocellular carcinoma and its possible association with prognosis Cancer 2002 94 1777 1786 10.1002/cncr.10402 11920541

[B41] Leung WK Ching AK Wong N Phosphorylation of caldesmon by PFTAIRE1 kinase promotes actin binding and formation of stress fibers Mol Cell Biochem 2010 350 201 206 21184254 10.1007/s11010-010-0699-8

[B42] Ai J Huang H Lv X Tang Z Chen M Chen T Duan W Sun H Li Q Tan R Liu Y Duan J Yang Y Wei Y Li Y Zhou Q FLNA and PGK1 are two potential markers for progression in hepatocellular carcinoma Cell Physiol Biochem 2011 27 207 216 10.1159/000327946 21471709

[B43] Mancone C Steindler C Santangelo L Simonte G Vlassi C Longo MA D’Offizi G Di Giacomo C Pucillo LP Amicone L Tripodi M Alonzi T Hepatitis C virus production requires apolipoprotein A-I and affects its association with nascent low-density lipoproteins Gut 2011 60 378 386 10.1136/gut.2010.211292 20940285

[B44] Hu SX Kyulo NL Xia VW Hillebrand DJ Hu KQ Factors associated with hepatic fibrosis in patients with chronic hepatitis C: a retrospective study of a large cohort of US patients J Clin Gastroenterol 2009 43 758 764 10.1097/MCG.0b013e31818be17c 19238091

[B45] Tseng HH Chang JG Hwang YH Yeh KT Chen YL Yu HS Expression of hepcidin and other iron-regulatory genes in human hepatocellular carcinoma and its clinical implication J Cancer Res Clin Oncol 2009 135 1413 1420 10.1007/s00432-009-0585-5 19387685 PMC12160254

[B46] Heim MH Innate immunity and HCV J Hepatol 2013 58 564 574 23063572 10.1016/j.jhep.2012.10.005

[B47] Moriishi K Mochizuki R Moriya K Miyamoto H Mori Y: Abe T Murata S Tanaka K Miyamura T Suzuki T Koike K Matsuura Y Critical role of PA29gamma in hepatitis C virus-associated steatogenesis and hepatocarcinogenesis PNAS USA 2007 104 1661 1666 10.1073/pnas.0607312104 17234812 PMC1776165

[B48] García-Mediavilla MV Pisonero-Vaquero S Lima-Cabello E Benedicto I Majano PL **Liver X receptor** α-**mediated regulation of lipogenesis by core and NS5A proteins contribute to HCV**-**induced liver steatosis and HCV replication** Lab Invest 2012 92 1191 1202 10.1038/labinvest.2012.88 22641099

[B49] Nguyen H Sankaran S Dandekar S Hepatitis C virus core protein induces expression of genes regulating immune evasion and anti-apoptosis in hepatocytes Virology 2006 354 58 68 10.1016/j.virol.2006.04.028 16876223

[B50] Schmitz KJ Wohlschlaeger J Lang H Sotiropoulos GC Malago M Steveling K Reis H Cicinnati VR Schmid KW Baba HA Activation of the ERK and AKT signaling pathway predicts poor prognosis in hepatocellular carcinoma and ERK activation in cancer tissue is associated with hepatitis C virus infection J Hepatol 2008 48 83 90 10.1016/j.jhep.2007.08.018 17998146

[B51] Lin W Tsai WL Shao RX Wu G Peng LF Barlow LL Chung WJ Zhang L Zhao H Jang JY Chung RT Hepatitis C virus regulates transforming growth factor beta1 production through the generation of reactive oxygen species in a nuclear factor kappaB-dependent manner Gastroenterology 2010 138 2509 2518 10.1053/j.gastro.2010.03.008 20230822 PMC2883661

[B52] Hwang-Verslues WW Sladek FM HNF4α - role in drug metabolism and potential drug target? Curr Opin Pharmacol 2010 10 698 705 10.1016/j.coph.2010.08.010 20833107 PMC2981672

[B53] Gautier-Stein A Zitoun C Lalli E Mithieux G Rajas F Transcriptional regulation of the glucose-6-phosphatase gene by cAMP/vasoactive intestinal peptide in the intestine. Role of HNF4alpha, CREM, HNF1alpha, and C/EBPalpha J Biol Chem 2006 281 31268 31278 10.1074/jbc.M603258200 16893891

[B54] Hertz R Seckbach M Zakin MM Bar-Tana J Transcriptional suppression of the transferrin gene by hypolipidemic peroxisome proliferators J Biol Chem 1996 271 218 224 10.1074/jbc.271.1.218 8550563

[B55] Sumi K Tanaka T Uchida A Magoori K Urashima Y Ohashi R Ohguchi H Okamura M Kudo H Daigo K Maejima T Kokima N Sakakibara I Jiang S Hasegawa G Kim I Osborne TF Naito M Gonzalez FJ Hamakubo T Kodama T Sakai J Cooperative interaction between hepatocyte nuclear factor 4α and GATA transcription factors regulates ATP-binding cassette sterol transporters ABCG5 and ABCG8 Mol Cell Biol 2007 27 4248 4260 10.1128/MCB.01894-06 17403900 PMC1900057

[B56] Ioannou GN Dominitz JA Weiss NS Heagerty PJ Kowdley KV Racial differences in the relationship between hepatitis C infection and iron stores Hepatology 2003 37 795 801 10.1053/jhep.2003.50147 12668972

[B57] Samantray J Zambare S Seyoum B Abou-Samra AB Glucose control and lipid metabolism in African American patients with type 2 diabetes mellitus and chronic hepatitis C viral infection Endocr Pract 2011 17 363 368 10.4158/EP10175.OR 21134881

[B58] Chellappa K Jankova L Schnabl JM Pan S Brelivet Y Fung CL-S Chan C Dent OF Clarke SJ Robertson GR Sladek FM **Src tyrosine kinase phosphorylation of nuclear factor HNF4**α **correlates with isoform**-**specific loss of HNF4**α **in human colon cancer** PNAS 2012 109 2302 2307 10.1073/pnas.1106799109 22308320 PMC3289305

[B59] Hatziapostolou M Polytarchou C Aggelidou E Drakaki A Poultsides GA Jaeger SA Ogata H Karin M Struhl K Hadzopoulou-Cladaras M Ilipopulos D An HNF4α-miRNA inflammatory feedback circuit regulates hepatocellular carcinoma Cell 2011 147 1233 1247 10.1016/j.cell.2011.10.043 22153071 PMC3251960

[B60] Bonzo JA Ferry CH Matsubara T Kim JH Gonzalez FJ **Suppression of hepatocyte proliferation by hepatocyte nuclear factor 4**α **in adult mice** J Biol Chem 2012 287 7345 7356 10.1074/jbc.M111.334599 22241473 PMC3293558

