# Supplementary material for: Quantitative proteomic analysis in HCV-induced HCC reveals sets of proteins with potential significance for racial disparity
Source: J Transl Med. 2013 Oct 1;11:239. doi: 10.1186/1479-5876-11-239 (PMC3850534; doi:10.1186/1479-5876-11-239)
Supplement: Additional file 2: Table S1 — Clinical characteristics of the study population. [file 1479-5876-11-239-S2.docx]

**Table S1**

Clinical characteristics of the study population

|  | Caucasian American (CA) | | | African American (AA) | | |
| --- | --- | --- | --- | --- | --- | --- |
| Variables | Normal  (HCV-) | CIR /  HCV+ | HCV+/  HCC+ | Normal  (HCV-) | CIR/  HCV+ | HCV+/  HCC+ |
| Age, years, mean (range) | 48.5  (38-64) | 51  (32-61) | 55.3  (48-67) | 42.3  (38-50) | 48.3  (33-62) | 59.3  (56-66) |
| Male sex, n (%) | 4 (67) | 4 (57) | 5 (83) | 3 (100) | 3 (67) | 3 (67) |
| Laboratory values, mean (SD) |  |  |  |  |  |  |
| ALBUMIN(g/dL) | NA | 3.1(0.7) | 2.77(0.3) | NA | 2.6(0.5) | 1.6(0.6) |
| AST(U/L) | NA | 47(14.4) | 91(104) | NA | 75.6(94) | 88(10) |
| ALT(U/L) | NA | 27(1.5) | 64(86.3) | NA | 51(5.4) | 66 (4.6) |
| BIL-T(mg/dL) | NA | 4(1.7) | 2.1(1.1) | NA | 5.3(3.1) | 2.2(0.8) |
| HEMOGLOBIN | NA | 9.1(1.8) | 13.9(1.7) | NA | 9.3(2.1) | 12(1.8) |

CIR= Cirrhotic; HCC = Hepatocellular carcinoma
